# Healthy individuals genetically at-risk for the development of Pemphigus vulgaris or Alopecia areata share disease-like cytokine dysregulation

**DOI:** 10.3389/fimmu.2024.1500284

**Published:** 2025-01-13

**Authors:** Rebekah R. Schwartz, Kristina Seiffert-Sinha, Animesh A. Sinha

**Affiliations:** Department of Dermatology, Jacobs School of Medicine and Biomedical Sciences, University at Buffalo, Buffalo, NY, United States

**Keywords:** pemphigus, alopecia, cytokine, HLA, Th17, Th2, autoimmunity

## Abstract

Autoimmune diseases (AID) are defined by immune dysregulation characterized by specific humoral and/or cell mediated responses directed against the body’s own tissues. Cytokines in particular play a pivotal role in the pathogenesis of AID, with proinflammatory cytokines contributing to the initiation and propagation of autoimmune inflammation, whereas anti-inflammatory cytokines facilitate regression of inflammation and recovery from acute phases of the disease. Parallel work by our group evaluating a comprehensive set of pro- and anti-inflammatory serum cytokines in Pemphigus vulgaris (PV) as well as Alopecia areata (AA) uncovered a similar pattern of inheritance specific immune dysregulation in these two distinct autoimmune skin diseases. In AA, we found healthy control subjects who are blood related to AA patients exhibit the same cytokine dysregulation in Th1 and Th17 pathways as do patients with AA. In PV, patients as well as individuals who are healthy but yet carry certain PV-associated HLA alleles (termed here as HLA-matched controls) share a similar, but not fully overlapping pattern of cytokine expression that is distinct from control subjects who do not type for these HLA alleles. Specifically, PV patients as well as HLA-matched controls demonstrate immunological activation of several pro-inflammatory-, Th17-, Th2-pathway associated cytokines, and the chemokine IL-8. Thus, in both AA and PV, we reveal cytokine dysregulations that are linked to genetic background. The presence of disease promoting pathways in not only patients, but also genetically related, but healthy control individuals further evokes the novel hypothesis that there may be co-existing disease counteracting immune protective mechanisms at play in thwarting the threat of disease in genetically predisposed individuals who, despite harboring disease associated immune imbalances, remain healthy. Our data underscore the known tendency of AID to cluster in families and support the notion of the shared genetic/common cause hypothesis across multiple AID.

## 1 Introduction

Pemphigus vulgaris (PV) is a life-threatening chronic autoimmune blistering condition affecting both the skin and mucous membranes, caused by autoantibodies targeting desmosomal proteins, which results in acantholysis in the suprabasilar epidermis, ultimately leading to blistering of the skin and/or mucosa. In addition to this autoantibody(autoAb)-mediated epidermal damage, it is well established there is co-existing systemic and local inflammation driven by cytokine expression (1). Cytokine secretion, in particular, results from T cell activation resulting in increased tissue inflammation. T cells also promote the survival and differentiation of B cells, thus allowing for continued autoantibody production (2).

In PV, there is a strong genetic association with two specific HLA haplotypes - HLA-DR4 (DRB1*0402) and DR6 (DQB1*0503); approximately 80% of North American patients express one, or both of these alleles (3). It is important to note that the presence of these PV-associated susceptibility alleles is not exclusive to patients, as the vast majority of individuals expressing these alleles remain disease free. Thus, HLA genes are not in themselves sufficient to cause disease; additional genetic and environmental factors (most of which are yet to be identified) are required (4).

Alopecia areata (AA) is also autoimmune in nature, characterized by nonscarring hair loss. Clinical manifestation of disease ranges from small-well circumscribed patches of hair loss limited to the scalp to diffuse hair loss over the entire body (5, 6). The exact etiopathogenesis of AA remains unclear, but again, both genetic and environmental factors have been implicated (6–9).

AA has a prevalence of 1.7% in the general population (10). However, familial aggregation data in AA patients has demonstrated an estimated lifetime risk of AA in siblings to be 7.1%, 7.8% in parents of AA patients, and 5.7% in offspring of AA patients (11). Twin studies have found a concordance rate of 42-55% in monozygotic twins and 0-10% in dizygotic twins (12, 13). Certain HLA alleles have also been linked to AA pathogenesis, however, the association is not nearly as strong as in PV (6, 14).

The high concordance rates in identical twins in AA (12, 13) and the strong HLA-associations seen in PV (15), along with the known tendency of AID to cluster in families (16), provide strong support for a clear genetic undergirding operative in each of these conditions. We have previously reported on the concept of familial inheritance in AA (17–19) and in PV (20–22), and here directly compare the impact of genetic background on cytokine patterns relevant to these two diseases. For this comparison, we particularly draw on data from two previous studies by our group on AA (18) and PV (23) to gain insight into patterns of cytokine dysregulation that are shared in patients as well as disease free but at-risk individuals (genetically susceptible healthy subjects) and distinct from healthy individuals not carrying any known genetic risk elements.

A multitude of studies have looked specifically at cytokine dysregulation in PV and in AA. PV had generally been considered a T helper (Th)2 disease, while AA was seen as predominantly Th1 mediated (24, 25). Newer studies have also implicated activation of the Th17 cytokine profile in both diseases (1, 23, 24, 26–28). However, none of the previous studies stratified their control populations based on their status of blood relation (in the case of AA) or HLA haplotype (in the case of PV). Applying this stratification to the control groups we studied allowed us to isolate the impact of genetic background on key immune mechanisms relevant to disease pathogenesis.

## 2 Methods

This work provides a new interpretation of data based on two separate but parallel studies examining cytokine dysregulation in AA and PV, Van Acker et al. (18) and Schwartz et al. (23). In short, for AA, we analyzed a number of Th1 and Th17 related cytokines, including interleukin (IL)-1β, IL-6, IL-10, IL-17A, IL-21, IL-22, IL-23, interferon (IFN)γ and tumor necrosis factor (TNF)α by ELISA in 64 patients with a diagnosis of AA, 16 unaffected relatives of AA patients, and 16 unaffected non-relative control subjects. At the time of the initial study, our subgroups were classified according to the National Alopecia Areata Registry (29) as Alopecia Areata persistent (n=17), Alopecia Areata transitory (n=15), Alopecia Universalis (n=16), and Alopecia Totalis (n=16). None of the AA patients were receiving systemic immunomodulating treatments at the time of their blood draw. Statistical analysis was conducted using Kruskall-Wallis non-parametric comparison method for analyzing population means. For PV, we analyzed 20 cytokines across multiple T helper cell and pro-inflammatory pathways including IL-1α, IL-1β, IL-2, IL-4, IL-5, IL-6, IL-8, IL-9, IL-10, IL-12, IL-13, IL-15, IL-17, IL-21, IL-22, IL-23, TNFα, IFNγ, MCP-1, and Eotaxin by multiplexed bead array assays in 116 PV patients, 15 healthy control subjects that carry PV-associated HLA-alleles and 14 healthy controls that did not carry PV-associated HLA-alleles. Our cohort of PV patients was further broken down by disease activity (active disease (n=75) and remission (n=55)), disease phenotype (mucosal (n=48), mucocutaneous (n=48), cutaneous (n=3) and undetermined (n=4)), and therapy status (off therapy (n=40), minimal therapy (n=34) and more than minimal therapy (n=56)). Statistical analysis was conducted using heteroscedastic T-tests. For detailed methodologies, as well as additional demographic details, please see Van Acker et al. (18) and Schwartz et al. (23).

## 3 Results

### 3.1 Healthy relatives of AA patients and healthy controls carrying PV-associated HLA alleles exhibit cytokine dysregulation similar to their respective patient population

**Figure 1:**
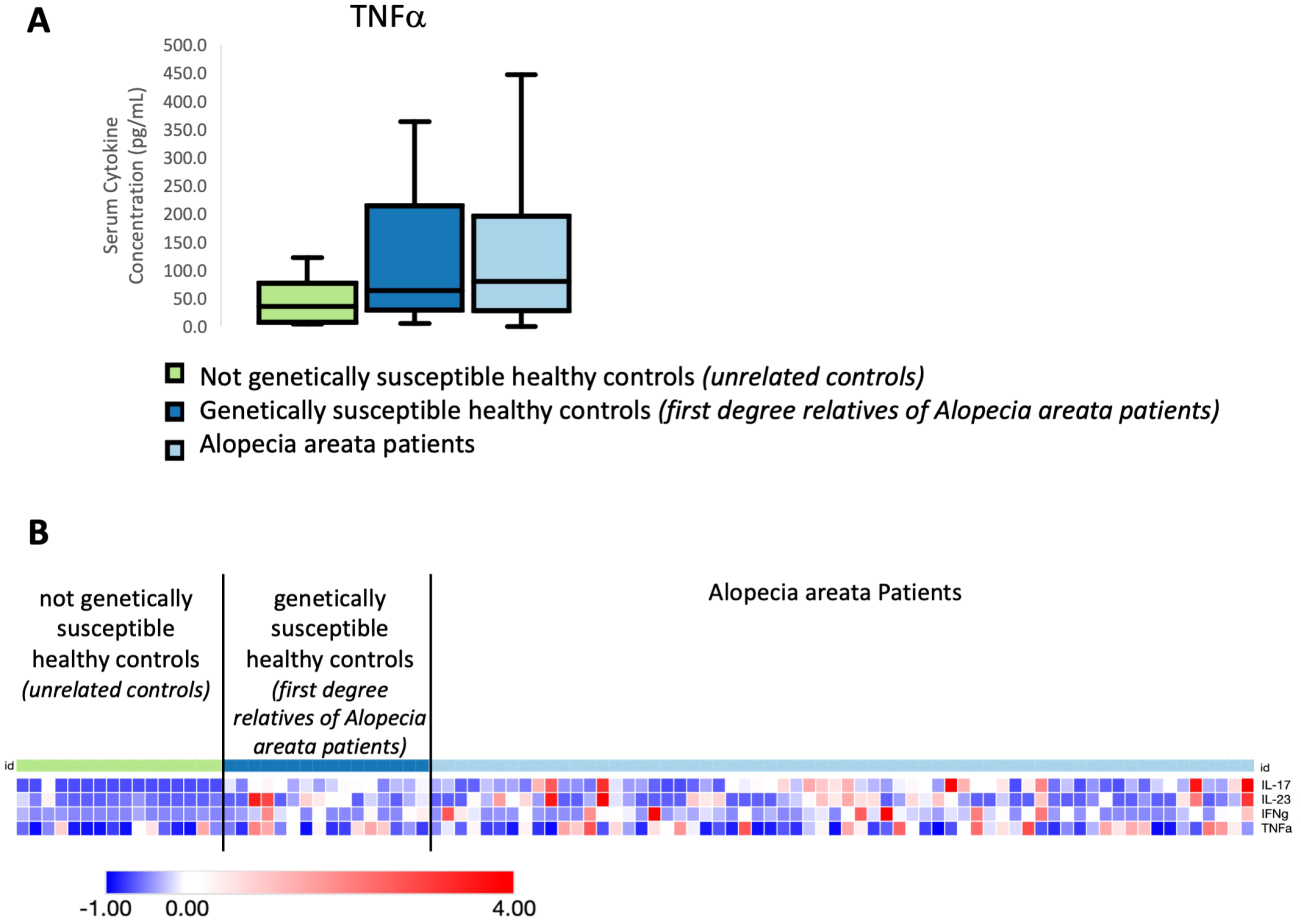
**(A)** Representative box and whisker plot of TNFα levels among Alopecia areata patients, genetically susceptible healthy controls (first degree relatives of Alopecia areata patients) and not genetically susceptible healthy controls (individuals with no family history of any autoimmune disease). **(B)** Heatmap of the distribution of four cytokines that were found to be significantly elevated in Alopecia areata patients and genetically susceptible healthy controls (first degree relatives of Alopecia areata patients) when compared to not genetically susceptible healthy controls (individuals with no family history of any autoimmune disease). Heatmap of z-score transformed data was created with Morpheus software. https://software.broadinstitute.org/morpheus.

In AA, including patients as well as healthy relatives and healthy non-relative control subjects, we found the Th1-associated cytokines IFNγ and TNFα and the Th17-associated cytokines IL-17A and IL-23 to be significantly upregulated in all AA patients in comparison to unaffected non-relatives (unrelated controls). **Figure 1A** shows a representative plot of these findings for TNFα. A heatmap representation of our z-score transformed data with supervised clustering shows that first degree relatives exhibit a marked increase in serum cytokine concentration similar to AA patients but distinct from controls not related to AA patients across multiple cytokines examined (**Figure 1B**). These data indicate that there is an inheritance-specific dysregulation within both the Th1- and Th17 cytokine pathways. No statistically significant differences were found when comparing subtypes of disease to one another. No significant differences were found for any of the other cytokines we measured.

**Figure 2:**
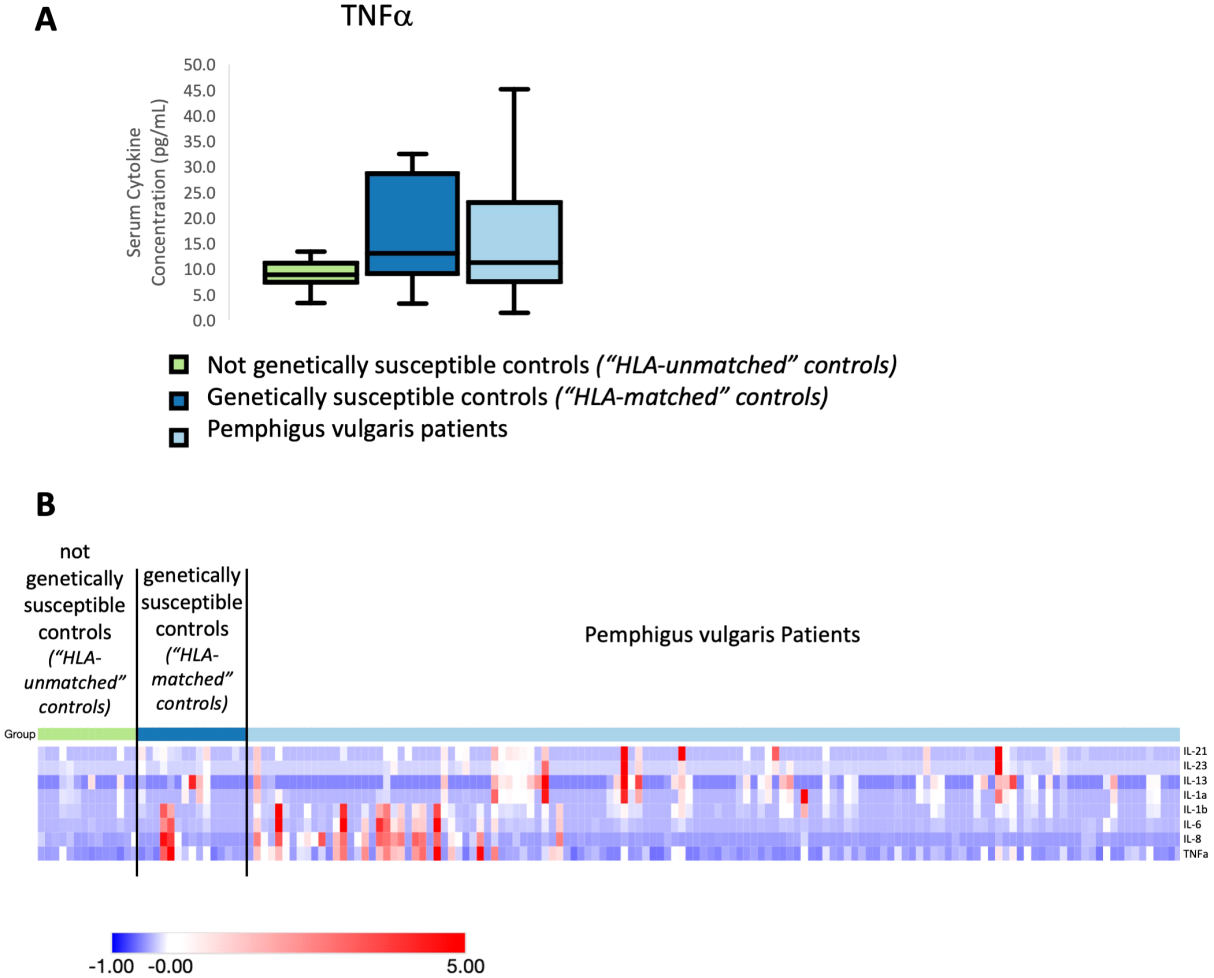
**(A)** Representative box and whisker plot of TNFα levels among PV patients, genetically susceptible healthy controls (“HLA-matched”) and not genetically susceptible (“HLA-unmatched”). **(B)** Heatmap of the distribution of cytokines that were found to be significantly elevated in Pemphigus vulgaris patients and genetically susceptible healthy controls (express Pemphigus vulgaris-associated HLA alleles) when compared to not genetically susceptible healthy controls (do not express Pemphigus vulgaris-associated HLA alleles). Heatmap of z-score transformed data was created with Morpheus software. https://software.broadinstitute.org/morpheus.

For PV, we uncovered a pattern mirroring what we observed in AA-genetically susceptible individuals, in this case linked to influence of HLA-association on cytokine expression. For a distinct set of cytokines (Th17: IL-21, IL-23; Th1: TNFα; Pro-Inflammatory: IL-1α, IL-1β, IL-6; Th2: IL-13; and chemokine: IL-8), we found that HLA-matched controls exhibited elevations in cytokines levels in a number of cases similar to patients. **Figure 2A** shows a representative plot of these findings for TNFα. **Figure 2B** displays these findings across multiple cytokines in heatmap format. While the mean levels of cytokines in HLA-matched controls were not quite as elevated as patients, we found no statistically relevant difference when comparing these two groups. However, healthy controls unmatched for HLA-susceptibility alleles had significantly lower cytokine levels when compared to patients. Thus, similar to our findings in AA, our data suggest an inheritance-specific dysregulation within numerous cytokine pathways (pro-inflammatory, Th2 and Th17) in healthy controls carrying genetic susceptibility for PV.

### 3.2 Inheritance linked cytokine patterns in PV and AA invoke novel concepts regarding autoimmune risk

We see similar patterns of genetically-based cytokine dysregulation in both AA and PV with at-risk individuals exhibiting cytokine concentrations in line with that seen in patients. Moreover, and notably, patients and at-risk individuals in both PV and AA share a dysregulation of IL-23 and TNFα across both diseases, supporting the notion of shared or common autoimmune pathomechanisms.

**Figure 3:**
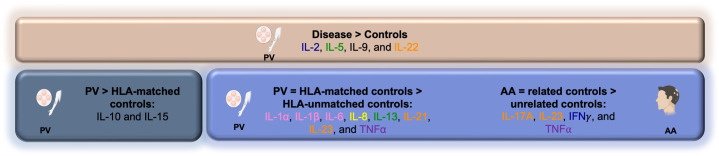
Summary schematic outlining cytokine dysregulation among PV and AA patients and control groups.

However, in addition to these shared cytokine changes in PV and AA, we also find dysregulation in other pathways and cytokines in PV - in particular, certain pro-inflammatory cytokines (IL-1α), chemokines (IL-8), and the Th2 pathway (IL-13) - that had not been analyzed in our AA studies. Of note, we found IL-2, IL-5, IL-22, and IL-9 to be significantly higher in PV patients compared to both HLA-matched controls and HLA-unmatched controls (**Figure 3**), reflecting changes unlinked to HLA type and suggesting cytokine activation is not strictly HLA driven, implicating the existence of additional genetic (and/or environmental) factors operative in patients.

Finally, we further postulated that due to the presence of AA/PV-associated cytokine profiles in genetically at-risk but healthy individuals, there may be mechanisms that actively counteract this (partial) autoimmune activation that operate to keep them disease free. In support of this hypothesis, we found certain cytokines such as IL-10 and IL-15 to be downregulated in HLA-matched healthy controls when compared to PV patients (**Figure 3**), yet they are not downregulated in non-HLA matched healthy controls compared to PV patients. These data suggest that a downregulation of certain cytokines may serve as one component of a necessary neutralizing force aimed at mitigating the existing partial (auto)immune activation seen in genetically susceptible, but healthy controls.

## 4 Discussion

The pathogenesis of both PV and AA is certainly multifactorial, influenced by a combination of patient genetics as well as environmental factors that ultimately lead to immune dysregulation. Highlighting cytokines as key effectors of the immune cascade, a multitude of studies in both conditions have attempted to identify the extent of dysregulation of numerous cytokines with potential relevance to disease pathogenesis (summarized in **Table 1**). A majority of studies indicate that AA is Th1- and Th17-driven disease (28), whereas PV appears to be driven by dysregulations in the Th2 and Th17 pathways (25). In accordance with previous studies, we also found find strong evidence for the activation of these pathways in PV and AA patients (**Table 1**).

**Table 1:** Comprehensive literature review of serum cytokine changes in AA and PV.

Pathway	Cytokine	AA Literature			PV Literature			Our AA Data	Our PV Data
# of studies showing:		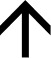	No Change	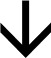	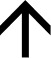	No Change	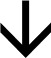		↑: PV > all CR 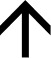 : PV > UMCR ⇧: PV > MCR
**Th17**	IL-17	13 (34–46)	3 (47–49)	–	8 (1, 50–56)	3 (57–59)	1 (60)	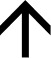	No change
	IL-21	2 (36, 37)	–	–	2 (27, 61)	2 (54, 57)	1 (62)	No change	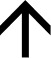
	IL-22	1 (36)	2 (42, 48)	–	1 (54)	–	1 (63)	No change	↑ 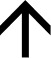 ⇧
	IL-23	2 (37, 64)	2 (40, 42)	–	2 (1, 65)	2 (51, 54)	1 (60)	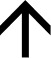	↑ 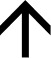
**Th1**	IFN-γ	13 (40, 42–45, 48, 66–72)	2 (37, 42)	–	6 (1, 52, 54, 58, 73–75)	4 (59, 76–78)	3 (79–81)	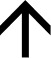	No change
	IL-2	8 (34, 35, 40, 45, 47, 66, 68, 71)	1 (48)	1 (44)	2 (54, 57)	6 (1, 51, 59, 76, 78, 82)	3 (74, 80, 81)	–	↑ 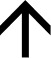 ⇧
	IL-12	2 (48, 66)	–	–	2 (52, 54, 59)	3 (1, 76, 77)	–	–	No change
Th2	IL-4	4 (43, 47, 71, 83)	3 (35, 48, 66)	1 (40)	7 (73, 78–81, 84–86)	5 (1, 54, 59, 62, 76, 82)	1 (74)	–	↑
	IL-5		1 (35)	2 (48, 87)	1 (54)	4 (1, 57, 59, 76)	–	–	↑ 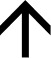 ⇧
	IL-13	3 (43, 45, 70)	2 (48, 66)	1 (42)	1 (54)	–	–	–	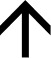
Pro-Inflammatory	TNFα	7 (35–37, 42, 64, 68, 69)	1 (66)	–	12 (54, 76, 77, 82, 88–96)	3 (51, 58, 97)	–	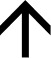	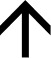
	IL-1	1 (42)	–	–	α: 7 (77, 89, 90, 92, 95, 96, 98) β: 7 (54, 57, 77, 89, 90, 95, 96, 98)	α: 4 (51, 54, 76, 97) β: 4 (1, 51, 76, 97)	β:1 (63)	–	α: 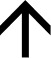 β: 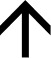
	IL-6	6 (36, 37, 44, 64, 66, 72)	1 (99)	–	10 (52, 59, 63, 75–77, 80, 82, 96, 97)	7 (51, 54, 57, 58, 60, 78, 91, 100)	–	No change	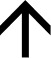
Other	IL-7	1 (101)	–	–	1 (57)	1 (76)	–	–	–
	IL-9	1 (43)	1 (66)	–	–	2 (1, 54)	–	–	↑ 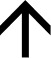 ⇧
	IL-10	3 (37, 40, 66)	4 (34, 45, 48, 99)	1 (102)	8 (51, 52, 54, 78, 81, 84–86, 103)	5 (1, 51, 52, 54, 58, 59, 76, 78, 81, 84–86, 92)	1 (74)	No change	↑ ⇧
	IL-15	2 (44, 47)	–	–	1 (104)	–	–	–	↑ ⇧
	IL-18	1 (105)	2 (48, 66)	–	–	–	–	–	–
	IL-25	1 (37)	–	–	–	–	–	–	–
	IL-31	1 (37)	–	–		–	–	–	–
	IL-33	1 (37)	–	–	1 (106)	–	–	–	–
	TGFβ	1 (42)	–	2 (35, 45)	2 (55, 92)	4 (1, 50, 76, 78)	3 (52, 60, 107)	No change	–
	IL-27	–	–	–	1 (91)	–	–	–	–
	CCL-11	–	–	–	–	–	1 (1)	–	–
	IL-36	–	–	–	1 (56)	–	–	–	–

The pathways currently considered most relevant in AA are indicated with bold text and pathways currently considered most relevant in PV are indicated with underlined text. Dashes indicate that no data is available. In our studies, cytokines were measured in pg/mL. ↑ indicates PV > all CR. 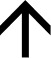
indicates PV > UMCR. ⇧ indicates PV > MCR.

Remarkably, in AA we report an inheritance-specific dysregulation of Th1 and Th17 pathway cytokines with an elevation in all AA clinical subtypes *as well as* first degree relatives in comparison to unrelated control subjects. Equally striking, we find healthy controls matched for the known PV-susceptibility alleles DRB1*0402 and/or DQB1*0503 (“HLA-matched”) exhibit an elevation of pro-inflammatory cytokines that is similar to that seen in PV patients when compared to HLA-unmatched controls (healthy controls who do not express the PV-associated susceptibility alleles) for Th2, Th17 and generally proinflammatory pathways. These data invoke a novel paradigm of disease susceptibility whereby unknown genetic elements in the case of AA or HLA molecules linked to PV susceptibility in the case of PV predispose individuals to a limited activation of inflammatory cytokines regardless of disease manifestation.

Thus, perplexingly, it appears that some genetically at-risk individuals, on the basis of their HLA status (PV) and/or other shared genetic background (AA), harbor disease-promoting inflammatory pathways, yet for reasons that are unclear, remain disease free. In support of this assumption, our group has previously found that in PV, HLA-matched controls share an upregulation of certain autoantibodies with patients when compared to HLA-unmatched controls (20). Furthermore, we found total antioxidant capacity to be similarly diminished in HLA-matched control and PV patients, but not in HLA unmatched controls (30). Thus, our work emphasizes that individuals who may have genetic susceptibility to these autoimmune skin diseases may be skewed towards activation of certain inflammatory pathways. On the other hand, it may be the case that HLA and/or other genetically driven differences in themselves are insufficient to produce the full scope of autoimmune dysregulation required to cross the threshold necessary to induce disease-specific phenotype. This idea is supported by the presence of cytokine dysregulations unique to PV patients alone, separate from HLA-matched control subjects (i.e. for IL-2, IL-5, IL-22, and IL-9). The latter findings indicate that cytokine dysregulation is not entirely HLA driven. Ultimately, disease manifestation is most likely to rely on the presence of additional genetic and/or environmental factors.

Genome-wide association studies (GWAS) may prove useful in identification of these additional genetic factors which contribute to disease. In PV, GWAS are limited, notably due to the low prevalence of the disease, however one study by Sarig et al. identified an association between the pro-apoptotic molecule ST18 and PV in a group of 100 Jewish PV patients and 400-age and population-matched controls (31). Little was reported on immune pathways within this work. In AA, however, GWAS have identified several immune-related pathways. In particular, literature has identified genes responsible for activation of IL-21 and IL-2, as well as the IL-2/IL-21, IL-2RA and HLA class II loci all to be implicated in AA (14, 32). This matches the Th1 and Th17 cytokine dysregulation we find in our work. However, GWAS have also found polymorphisms in the promotor region of IL-13 to be associated with AA (33).

Perhaps most intriguingly, a disease relevant, and at least partial, autoimmune activation in individuals that share genetic risk elements evokes a further novel hypothesis that there may be additional mechanisms at play which *prevent* these at-risk individuals exhibiting disease-like inflammation from progressing to frank clinical disease. It may be the case, for example, that specific counter-regulatory immune mechanisms offset the disease-driving cytokine dysregulation. One component of the necessary neutralizing force against the existing partial autoimmune activation seen in PV HLA-matched controls may be the downregulation of other specific cytokines, such as we observed for IL-10 and IL-15. Previous work in our lab supports this concept within both PV and AA. In gene expression studies in PV, our group has previously identified a specific ‘protection signature’ in HLA-matched controls, indicating that these individuals down- or up-regulate a specific set of genes that is otherwise similarly expressed in PV patients and healthy individuals not matched for PV-associated HLA alleles (HLA-unmatched controls) (22). Interestingly, a downregulation of the IL-13RA1 gene is part of this signature, supporting the importance of cytokine dysregulation in disease development or prevention thereof. Echoing this theme, previous gene microarray work in our lab uncovered specific “inheritance,” “disease,” and “severity” transcriptional signatures within AA patients, healthy relatives (related controls), and healthy non-relatives (unrelated controls) (19).

**Figure 4:**
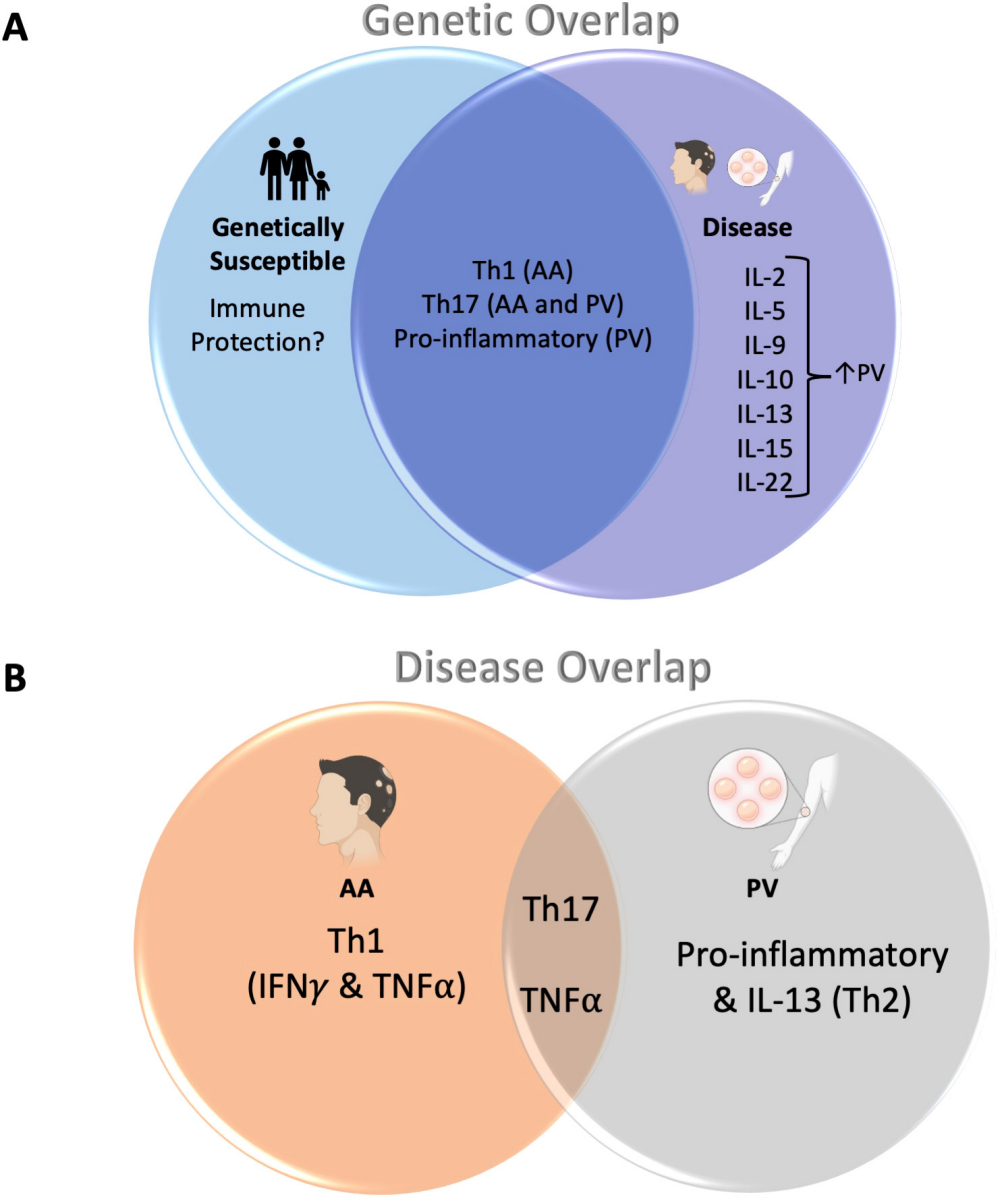
**(A)** Cytokine dysregulation shared across related controls/HLA-matched controls and patients with AA/PV: The overlapping portion of the Venn diagram shows cytokine pathways that are shared between both patients with AA and PV, as well as their genetically susceptible counterparts (related controls or HLA-matched controls, respectively). The portion to the right show cytokines that were found to be upregulated in disease alone (note: outside of IL-10 and IL-22, these cytokines were not analyzed for AA patients). **(B)** Cytokine dysregulation shared across both AA and PV: Both AA and PV share a dysregulation in Th17-pathway associated cytokines as well as TNFα (overlapping area of the Venn diagram). In addition, AA shows dysregulation in Th1-pathway cytokines IFNγ and TNFα (left). PV, on the other hand, shows an additional dysregulation in other pro-inflammatory cytokines (IL-1α, IL-1β, IL-6, IL-8) as well as the Th2 pathway cytokine IL-13 (right).

**Figure 4** summarizes our findings regarding these novel concepts regarding disease risk, and the immune requirements and limitations linked to disease development. Our data allow for a more complete and deeper understanding of how cytokine pathways are altered in PV and AA pathogenesis and outline a potential role for HLA haplotype/familial relation in disease.

In summary, our data uncover a genetic underpinning to Th1- and Th17-related cytokine dysregulation in AA and to Th2-, Th17- and pro-inflammatory cytokine dysregulation in PV that are not restricted to patients. Our work supports the novel hypothesis that healthy individuals related to AA patients as well as individuals genetically susceptible to PV exhibit disease-associated immune disturbances that in themselves fall below the threshold required for disease development, and/or possess counter-regulatory mechanisms that prevent progression to active disease.

Though the work presented here focuses solely on peripheral cytokine dysregulation in AA and PV, target tissues of these diseases such as hair follicles in AA and keratinocytes in PV may harbor their own genetic variations and respond to an altered cytokine milieu differently. Our studies do not shed light on the effect of peripheral cytokine dysregulation on these target tissues. More work is thus required to identify how and if the peripheral cytokine dysregulation we find ultimately affects target tissues, and whether the cytokine dysregulation in genetically susceptible but healthy controls would extend to target tissues. Such work would need to focus on both lesional and non-lesional skin in patients, whereas genetically susceptible individuals could provide only non-lesional skin samples for comparison.

Nevertheless, knowledge of the extent and limitations of genetic contribution to disease provides new details regarding the mechanistic road map underlying the development of AA and PV, and shines a spotlight on the murky and previously underrecognized and understudied question of why certain individuals develop disease while others do not. Moreover, our data add to the knowledge base of how and why particular autoimmune diseases tend to cluster within families in support of the common gene/cause hypothesis. This work provides a stepping stone for further investigation to the precise mechanisms that produce disease, and ultimately those that may prevent it as well. Future work will be necessary to pinpoint potential disease-counteracting immune mechanisms in genetically predisposed individuals that could inform an entirely new approach to disease management and therapeutics.

## Data Availability

The raw data supporting the conclusions of this article will be made available by the authors, without undue reservation.
